# Proteome Analysis of Pathogen-Responsive Proteins from Apple Leaves Induced by the Alternaria Blotch *Alternaria alternata*

**DOI:** 10.1371/journal.pone.0122233

**Published:** 2015-06-18

**Authors:** Cai-xia Zhang, Yi Tian, Pei-hua Cong

**Affiliations:** 1 Key Laboratory of Horticulture Crops Germplasm Resources Utilization, Ministry of Agriculture, P.R. China; 2 Research Institute of Pomology, Chinese Academy of Agricultural Sciences, P.R. China; The Chinese University of Hong Kong, HONG KONG

## Abstract

Understanding the defence mechanisms used by apple leaves against *Alternaria alternate* pathogen infection is important for breeding purposes. To investigate the ultrastructural differences between leaf tissues of susceptible and resistant seedlings, in vitro inoculation assays and transmission electron microscopy (TEM) analysis were conducted with two different inoculation assays. The results indicated that the resistant leaves may have certain antifungal activity against *A*. *alternate* that is lacking in susceptible leaves. To elucidate the two different host responses to *A*. *alternate* infection in apples, the proteomes of susceptible and resistant apple leaves that had or had not been infected with pathogen were characterised using two-dimensional electrophoresis (2-DE) and matrix-assisted laser desorption/ionisation time-of-flight tandem mass spectrometry (MALDI-TOF-TOF MS). MS identified 43 differentially expressed proteins in two different inoculation assays. The known proteins were categorised into 5 classes, among these proteins, some pathogenesis-related (PR) proteins, such as beta-1,3-glucanase, ascorbate peroxidase (APX), glutathione peroxidase (GPX) and mal d1, were identified in susceptible and resistant hosts and were associated with disease resistance of the apple host. In addition, the different levels of mal d1 in susceptible and resistant hosts may contribute to the outstanding anti-disease properties of resistant leaves against *A*. *alternate*. Taken together, the resistance mechanisms of the apple host against *A*. *alternate* may be a result of the PR proteins and other defence-related proteins. Given the complexity of the biology involved in the interaction between apple leaves and the *A*. *alternate* pathogen, further investigation will yield more valuable insights into the molecular mechanisms of suppression of the *A*. *alternate* pathogen. Overall, we outline several novel insights into the response of apple leaves to pathogen attacks. These findings increase our knowledge of pathogen resistance mechanisms, and the data will also promote further investigation into the regulation of the expression of these target proteins.

## Introduction

Plants are usually under numerous threats of pathogen infection, and some of them act as hosts to invasive pathogens [1, 2]. Host-pathogen interactions involve complicated defences, generally, host plants express a wide range of resistance-related proteins in response to pathogen attacks, include pathogenesis-related (PR) enzymes [3]. A comprehensive understanding of the proteins induced by pathogens will help reveal the complex molecular mechanisms that mediate plant disease resistance and will aid the development of new strategies to increase disease resistance in some economically important crops. Apple (*Malus domestica*) is considered a model fruit plant due to its world-wide economic importance; a large number of apple cultivars dominate world fruit production [4, 5]. However, to date, apple cultivation has been limited by many kinds of fungal diseases, and the domestic apple has become an important fruit crop in which to study commercial traits such as disease resistance [6, 7]. Among the many fungal diseases affecting apple trees, Alternaria blotch, which is caused by the pathogen *Alternaria alternata*, has been a destructive apple disease in China and other East Asian countries [8]. Currently, the disease is spreading worldwide and results in severe negative effects on apple production [9, 10]. *A*. *alternata* can cause circular blackish spots on apple leaves in late spring or early summer, resulting in serious defoliation and decreased fruit quality [11, 12]. Currently, management of the Alternaria blotch occurs mainly through traditional chemical control agents instead of resistant cultivars.

The secretion of specific sets of proteins has been known to play decisive roles in plant—fungus interactions. More recently, the significance of resistance-related proteins has been reported for many plant-pathogen interactions [13, 14], and some intensive attempts have also been made to engineer resistance to apple disease. Several genes have been isolated that are related to apple disease resistance [3, 15–18]. The functions of some PR enzymes, including chitinases, β-1,3-glucanases and peroxidases, which act directly against pathogens, were also confirmed by some assays [15–17]. Whereas previous studies on the interaction between plants and pathogenic microorganisms were focused largely on some model plants and pathogens, relatively few have been performed using apple and its pathogens.

Over the last few years, the use of proteomic analysis has drastically expanded for the identification of stress-related proteins based on two-dimensional electrophoresis (2-DE) and mapping the dynamics of differential expression involved in the host-plant response to biotic stresses, such as pathogen-crop interactions [19, 20].

In apple, the molecular mechanisms of disease resistance against *A*. *alternata* have not been illustrated clearly. Although most apple resistance genes have been identified [21], little is known about their biological roles. To date, very few proteomic analyses of apple host-pathogen interactions have been reported. Considering that Alternaria blotch is the most common disease influencing apple production, studies of the host self-defence mechanisms that closely relate to anti-disease properties should be performed. A recent study showed that susceptible and resistant apples exhibit different patterns of gene expression in response to *A*. *alternata* infection [7]. To understand the host defence mechanisms induced by the pathogen, we investigated the specific stress-related proteins that mediate interactions between *A*. *alternata* and its host and aimed to understand the molecular mechanisms of plant-pathogen interactions. The proteins identified as associated with the antifungal mechanisms of the host plant against *A*. *alternata* may be novel links to the antifungal mechanisms of resistant apples.

This study may provide clues to the molecular mechanisms of apple resistance to *A*. *alternata*, accelerating the process of apple molecular breeding and providing a theoretical basis and technical reference for future genetic improvement studies of high-quality, disease-resistant apple varieties.

## Materials and Methods

### Plant materials and fungal pathogen

The plant material used in this study came from an 8-year-old apple seedling population consisting of 110 seedlings derived from a cross of ‘Huacui’ and ‘Golden Delicious’, which was grown in an experimental orchard at the Institute of Pomology at the Chinese Academy of Agricultural Sciences (CAAS; Xingcheng, China). The evaluation of a disease rating by field inoculation was previously conducted on these 110 seedlings in 2008 and 2009 [7]. Based on these evaluation results, two of the 110 seedlings with obvious resistance differences, including one highly susceptible seedling and one highly resistant seedling, were chosen as hosts for this study.

The aggressive strain of *A*. *alternata* used in this study was provided by the Fruit Plant Protection Research Center at the Institute of Pomology at CAAS. To culture this fungal strain for in vitro assays, *A*. *alternata* mycelium was incubated on petri plates containing potato dextrose agar (Sigma Chemical Co., St. Louis, MO) at 25°C to harvest spores.

### In vitro inoculation assays of the pathogen on host plants

The inoculation assays were performed as described by [8] with some modifications. The samples were harvested 48 h after inoculation; both infected and control samples were harvested at the same time after inoculation. Three replicates for each treatment were performed, and each replicate contained 30 leaves. The entire experiment was repeated three times to ensure reliable results. Samples were harvested, immediately frozen in liquid nitrogen, and then ground to a fine powder prior for protein extraction.

### Transmission electron microscopy (TEM) analysis

The selected control and inoculated leaves were sampled and collected 48 h after inoculation. The samples were cut into 2 to 3 mm^2^ pieces, fixed in 3% glutaraldehyde in 0.1 M sodium phosphate buffer (pH 7.0) at 4°C for 24 h, washed 3 times in 0.1 M sodium phosphate buffer, post-fixed in 1% OsO_4_ in 0.1 M sodium phosphate buffer for 4 h at 4°C, and then washed in 0.1 M sodium phosphate buffer. Following dehydration in a graded acetone series, the samples were embedded in Epon-Araldite-DDSA and sectioned at 2 μm thickness. Sections on grids were stained with lead citrate for 2.5 min before observation by TEM (Hitachi H-7500, Hitachi Co. Ltd., Tokyo, Japan).

### Protein extraction and 2-DE

The protein extraction was performed as described by [22] and [23] with some optimisation. In short, 2 g of frozen lyophilised tissue powder was resuspended in 4 mL of ice-cold extraction buffer (30% sucrose w/v, 20 mM Tris-HCl (pH 8.0), 10 mM EGTA, 1 mM DTT, 1% Triton X-100 v/v, 2% β-mercaptoethanol v/v, and 1 mM PMSF). After the sample was vortexed for 10 min at room temperature, an equal volume of precooled Tris—HCl (pH 7.5)-saturated phenol was added, and then the mixture was further vortexed for 20 min. After centrifugation (15,000 *g* at 4°C for 20 min), the upper phase was collected and transferred to a new centrifuge tube. Proteins were precipitated from the phenol phase with three volumes of 100 mM ammonium acetate in methanol overnight at -20°C. The protein pellets were subsequently rinsed three times with cold acetone containing 13 mM DTT. After centrifugation, the rinsed pellets were air-dried and resuspended in lysis buffer [7 M urea, 2 M thiourea, 4% (w/v) CHAPS, 0.5% (v/v) IPG buffer, and 1% (w/v) DTT]. The protein solution was either used immediately for 2-D electrophoresis or maintained at -80°C prior to use. The protein concentration was determined using a 2-D Quant Kit (GE Healthcare).

2-DE was performed according to [22] with some modifications. A sample containing 800 μg of total protein was loaded onto an immobilised pH gradient (IPG) strip (18 cm, pH 4–7 linear, GE Healthcare) and rehydrated for 12 h at room temperature. Then, the strips were subjected to isoelectric focussing (IEF) in an Ettan IPGphor system according to the following procedure: 300 V for 1 h, 600 V for 1 h, 1,000 V for 1 h, 5,000 V for 1 h, and 10,000 V for 6 h. After IEF, the strips were transferred to perform the SDS-PAGE or were stored at -20°C. Prior to the second dimension analysis, the strips were equilibrated for 15 min in 10 mL of equilibration solution (50 mM Tris pH 8.8, 6 M urea, 30% glycerol, 2% SDS, and 0.002% bromophenol blue) containing 1% DTT w/v, followed by incubation in 4% iodoacetamide w/v in the same solution for 15 min. The separation of proteins in the second dimension was performed using SDS polyacrylamide gels (12.5%) on the Ettan DALT System (GE Healthcare): 0.5 w / gel for 30 min and 10 w/ gel for 5 h. After electrophoresis, the gels were stained with Coomassie Brilliant Blue (CBB) R-350.

### Image and data analysis

The 2-D gels were scanned at a resolution of 600 dpi, and the image analysis was conducted using Image Master 2D Platinum Version 7.0 software (GE Healthcare). The *M*_r_ of each protein spot in the gel was determined by referencing protein markers. For each treatment, three images were obtained, representing 3 independent biological replicates, and these replicates were grouped as a class to calculate the average volume of all protein spots. The standard values of protein spots on the three replicate 2D gels from each treatment were exported to SPSS Version 13.0 (Lead Technologies, Chicago, Illinois, USA) for statistical analysis. Only those with significant and consistent changes were counted as differentially accumulated proteins (>1.5-fold, p<0.05).

### In-gel digestion and protein identification

Proteins were identified basing on the method of [24] with minor modifications. In brief, protein spots of interest were manually excised from the 2-DE gels and destained for 1 h at room temperature using a freshly prepared wash solution of 30% acetonitrile (ACN) and 100 mM ammonium bicarbonate (1:1 v/v). The wash solution was removed, and the spots were minced and lyophilised. The proteins were digested using a trypsin solution (10 ng/μL sequencing-grade modified trypsin in 50 mM ammonium bicarbonate), and the samples were swollen at 4°C for 30 min. The protein spots were then placed in a 37°C incubator overnight. After digestion, the peptides were collected, and the gel pieces were washed three times with 0.1% trifluoroacetic acid (TFA) in 60% ACN to collect the remaining peptides. The peptides were desalted using ZipTipC 18 pipet tips (Millipore, Bedford, USA). For matrix-assisted laser desorption/ionisation time-of-flight tandem mass spectrometry (MALDI-TOF-TOF/MS), the peptides were eluted onto the target plate with an equal volume of a freshly prepared 5 mg/ml solution of 4-hydroxy-α-cyano-cinnamic acid in 50% (v/v) ACN containing 0.1% TFA. The samples were analysed on a 4800 Plus MALDI TOF/TOF TM Analyser (Applied Biosystems, USA). The data were searched with GPS Explorer (GPS Explorer TM software, Applied Biosystems, USA) using MASCOT (Matrix Science, London, UK) as a search engine. The parameters were set as follows: the database was NCBI (nr); taxonomy was set to Viridiplantae (Green Plant); max missed cleavages was set to 1; and the enzyme was set to trypsin. The quality error scope setting was 0.4 Da. The unmentioned parameters were set according to their default values in the software. Some other criteria were also considered in the final identification results, including the number of matching peptides, the molecular weight (MW) of the protein and the isoelectric point (PI) of the protein. To determine the confidence of the identification results, the results with protein scores above 100 and protein score C.I. percentages above 95% were chosen as positive. Only the best matches with high confidence levels were selected.

## Results

### In vitro inoculation assays and microscopic observation

Previous research by the current authors reported the dynamic changes of *A*. *alternata*-infected susceptible host leaves [25]. Thus, to investigate the ultrastructural differences between leaf tissues of susceptible and resistant seedlings, in vitro inoculation assays and TEM analysis were conducted in two different host populations.

**Fig 1:**
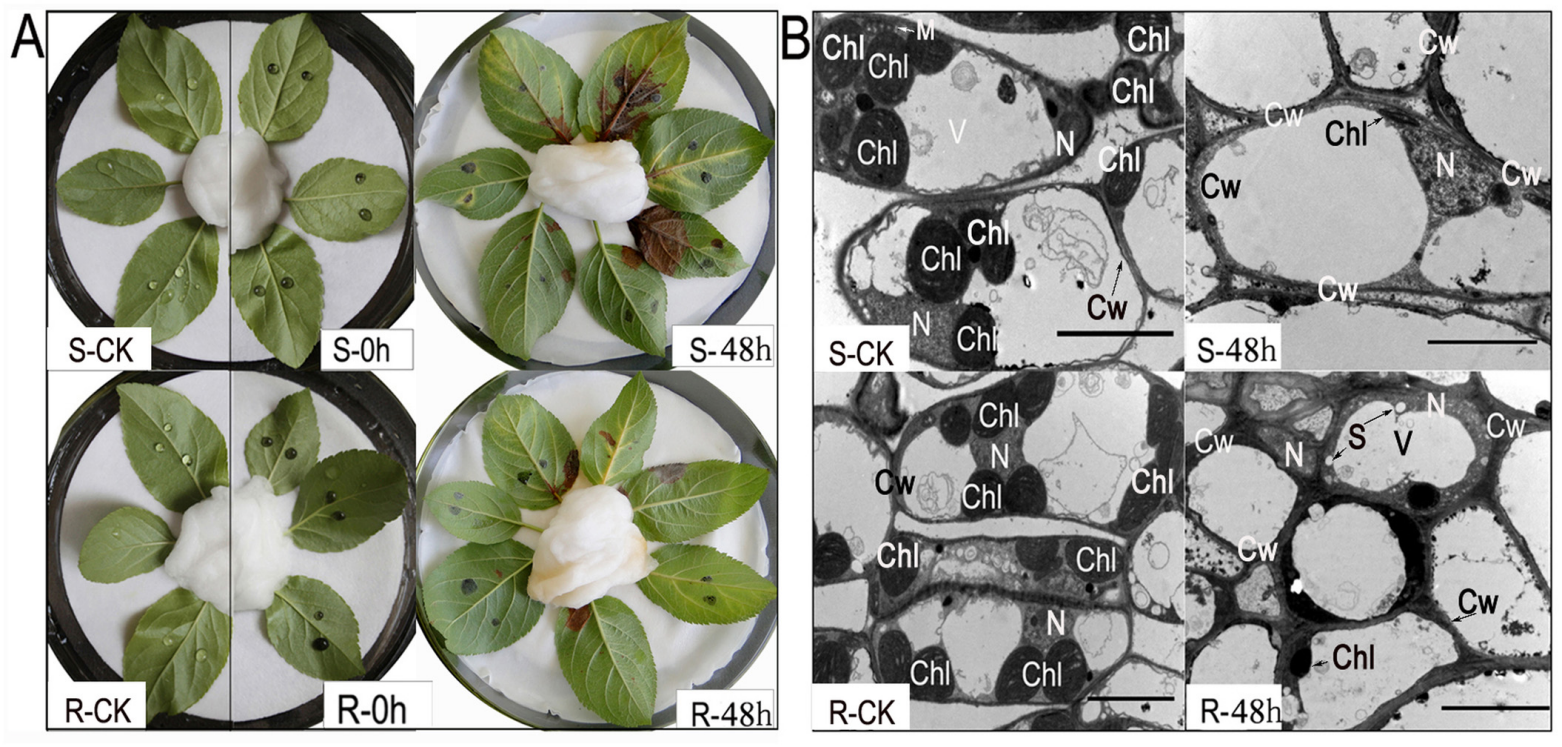
Reactions of apple leaves toward the fungal pathogen *A*. *alternata*. A: Observation of the reactions of resistant (R) and susceptible (S) leaves 0 h and 48 h after inoculation. CK: The control samples were treated with water instead of inoculums. B: Relative changes in symptoms of infected host cells before and after treatment with pathogen visualised by transmission electron micrographs (Bars = 5 μm) Chl: Chloroplast; V: Vacuole; Cw: Cell wall; N: Nucleus; S: Starch grains.

As shown in Fig 1A, 48 h after inoculation, the susceptible (S-48 h) and resistant leaves (R-48 h) exhibited differences compared with the controls; some obvious disease symptoms appeared on the susceptible leaves (S-48 h), and only a few symptoms appeared on the resistant leaves (R-48 h). To better understand the intrinsic molecular changes that occurred during the inoculation assays, we conducted TEM analysis of these leaves. As demonstrated in Fig 1B, few obvious differences were observed between susceptible (CK-S) and resistant (CK-R) control leaves; some basic cell structures were observed including chloroplasts (Chl), vacuoles (V), cell walls (Cw), nuclei (N) and starch grains (S). However, 48 h after inoculation, the cellular structures of the test tissues were significantly diminished. The susceptible tissues suffered more severe destruction than the resistant ones, with few identifiable structures remaining other than some reserved cell wall (Fig 1B). Based on these evaluations of susceptible and resistant seedlings, the harvest times for the leaves were set at 0 and 48 h.

### Comparison of proteome expression induced by the *A*. *alternata* pathogen in resistant (R) and susceptible (S) leaves

**Fig 2:**
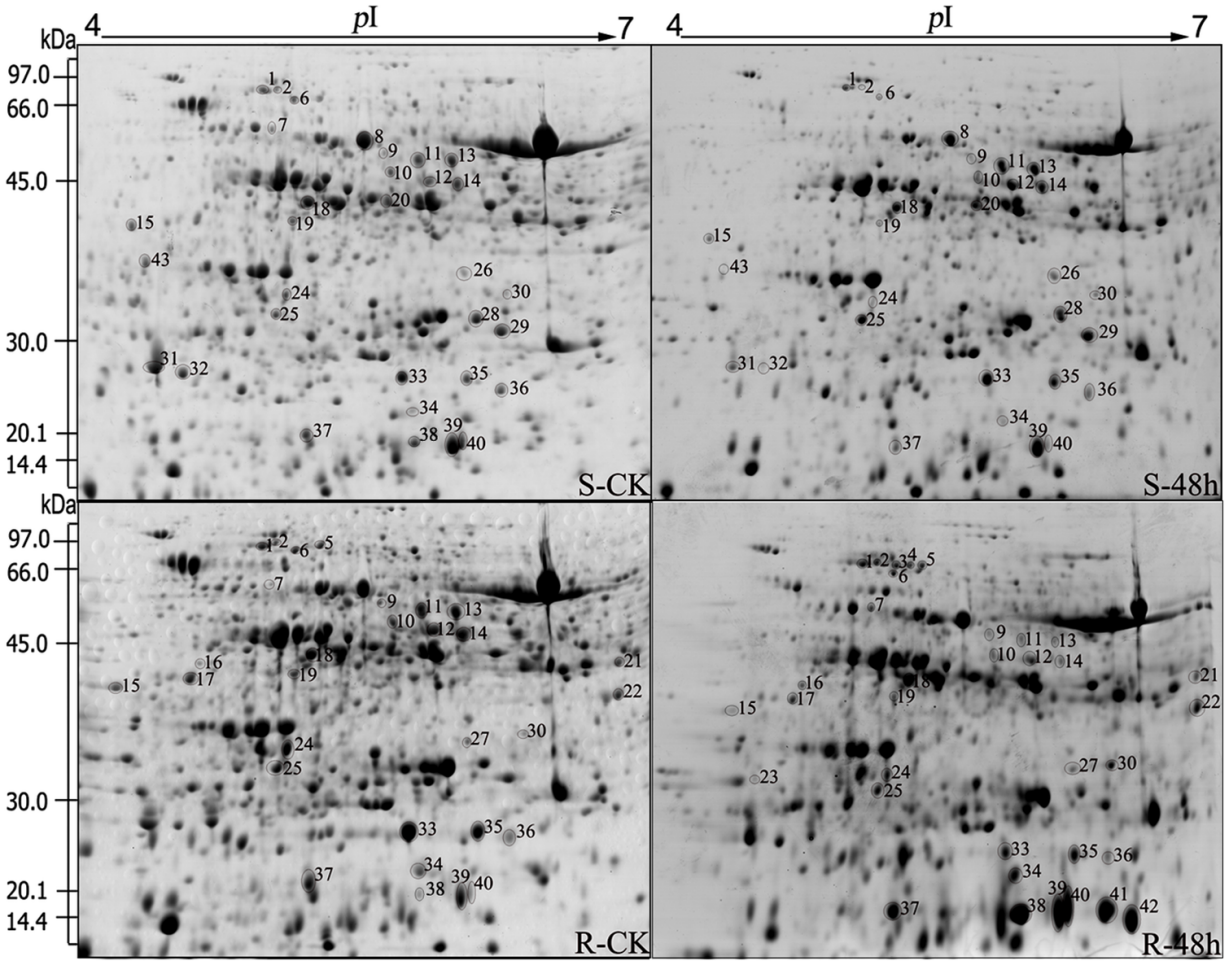
2-DE analysis of proteins induced by the *A*. *alternata* pathogen in resistant (R) and susceptible (S) leaves. Total protein (800 μg) was separated on 2D gels (pH 4–7) and stained with CBB R-350. Approximate molecular masses and pIs are indicated in the margins. Circles indicate the 43 proteins identified by MALDI-TOF-TOF/MS that changed in abundance more than 1.5-fold between controls and treated samples. The affected proteins are numbered, and the numbers correspond to the numbers in Fig 3. This figure represents three biological replicates.

**Fig 3:**
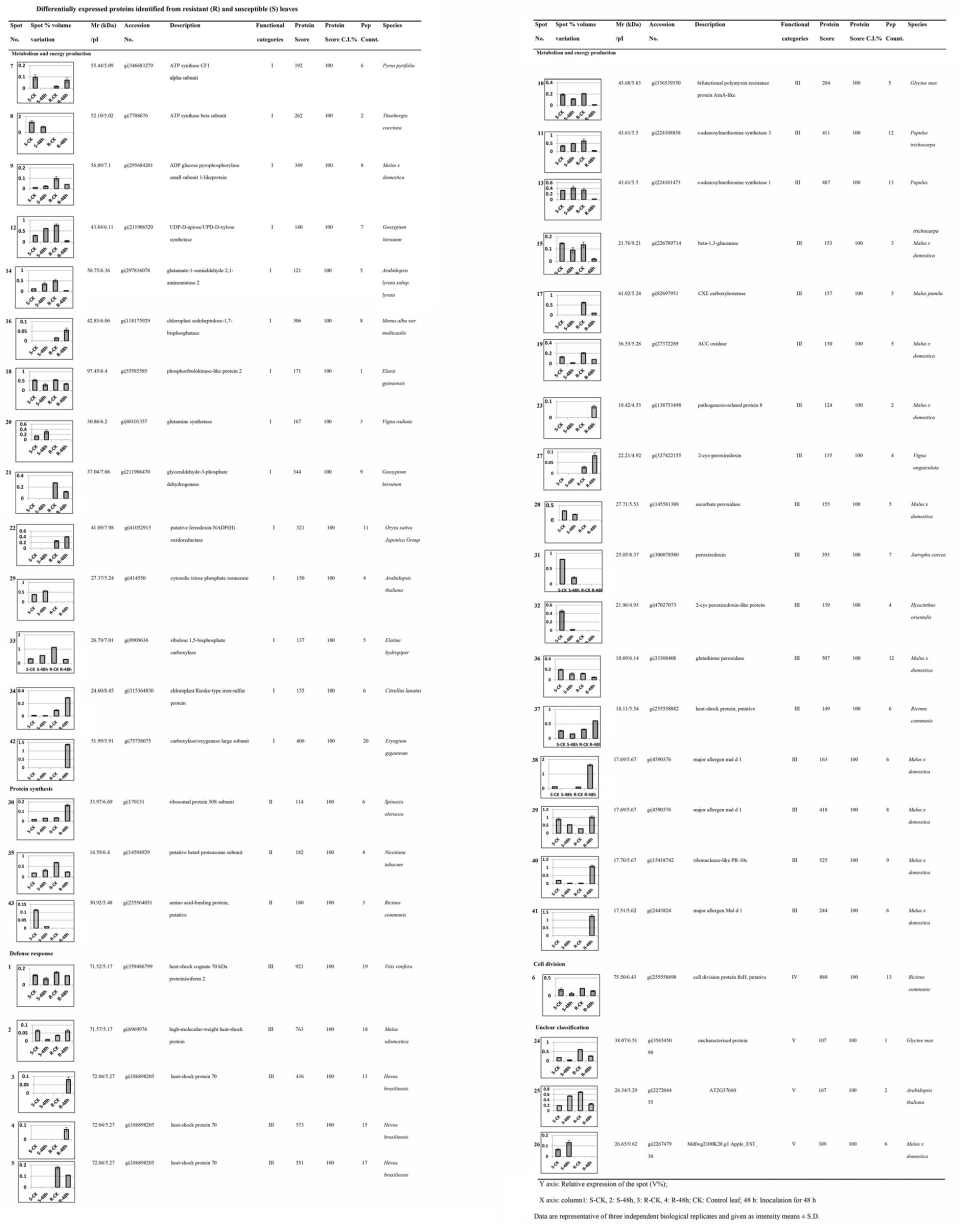
Differentially expressed proteins identified from resistant (R) and susceptible (S) leaves.

**Fig 4:**
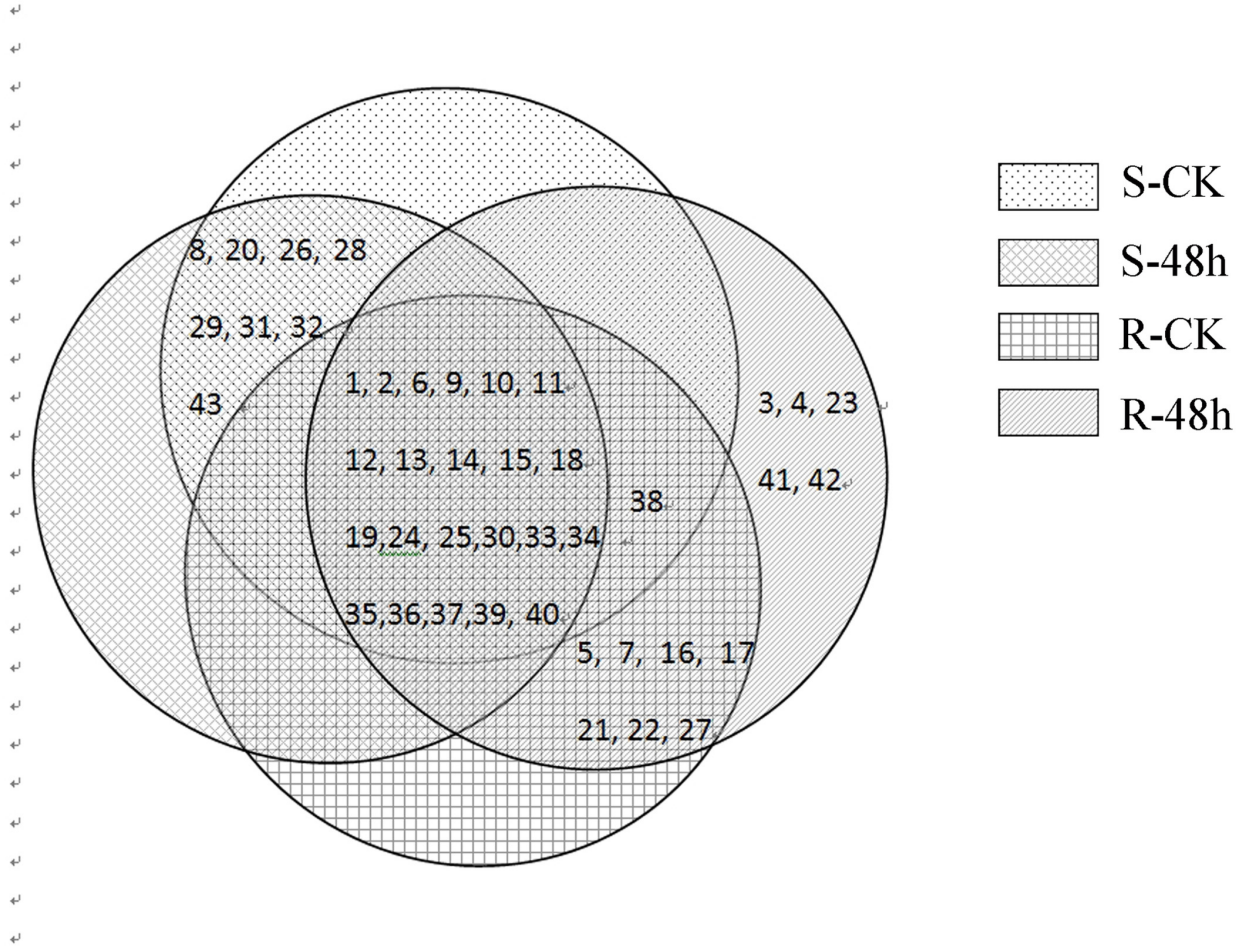
Venn diagram showing the distribution of protein spots expressed as indicated in Fig 2.

We next explored changes in total protein expression induced by the *A*. *alternata* pathogen in resistant and susceptible leaves. To this end, the control and infected samples were prepared and subjected to 2-DE electrophoresis. The electrophoresis results revealed that approximately 850 protein spots were detected in each sample (Fig 2). Compared with their respective controls, two different inoculation assays resulted in a total of 43 protein spots that were differentially expressed in inoculated samples by at least 1.5-fold (Fig 2). Of these protein spots, 22 proteins were significantly induced both in resistant and susceptible leaves and were observed in every sample, including S-CK, S-48 h, R-CK and R-48 h. To demonstrate the distribution of differentially expressed proteins across the different samples, all protein spots are shown in a Venn diagram using numbers taken from Figs 2 and 3 (Fig 4). As seen in Fig 4, in addition to the 22 spots common to all samples, 8 spots (spots no. 8, 20, 26, 28, 29, 31, 32, and 43) were differentially expressed in the control and treated susceptible leaves (S-CK and S-48 h), whereas 13 spots (spots no. 3, 4, 5, 7, 16, 17, 21, 22, 23, 27, 38, 41, and 42) were induced only in resistant leaves (R-CK and R-48 h).

### Protein identification and functional categorisation

Based on the criteria described in the methods section, 43 spots were successfully identified from the two inoculation assays. According to the identification results, approximately 13 proteins were highly matched with proteins from the *Malus x domestica* species, and the remaining 30 proteins were identified from other databases. Among these differentially expressed proteins, 12 and 16 proteins were up-regulated in susceptible and resistant leaves, respectively, whereas 20 and 19 proteins were down-regulated in susceptible and resistant leaves, respectively (Fig 3). The identified proteins induced by the pathogen are listed in Fig 3.

**Fig 5:**
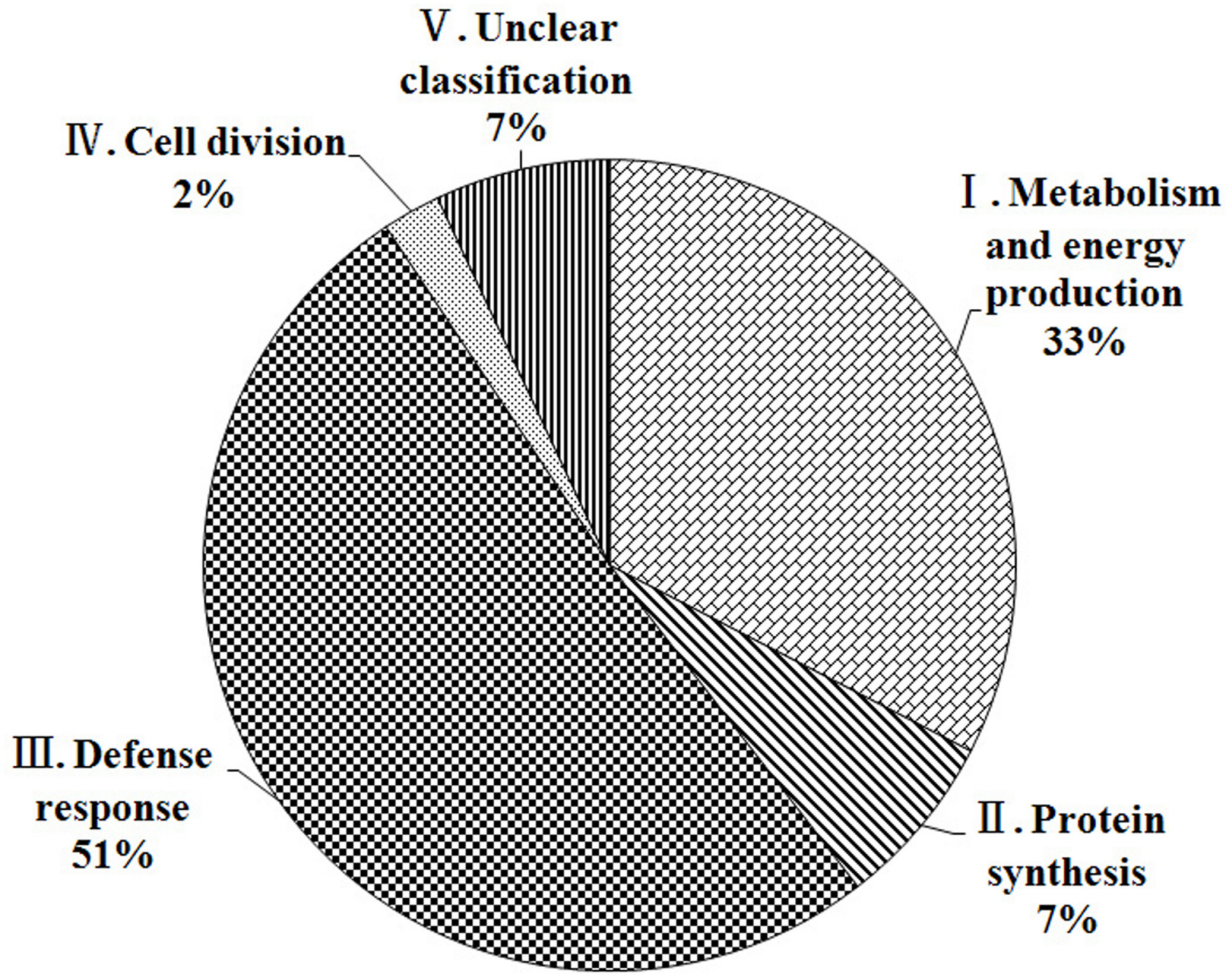
Functional categorisation of the identified proteins that are differentially regulated in resistant (R) and susceptible (S) leaves infected with the *A*. *alternata* pathogen. A total of 43 identified proteins were assigned to the functional categories. The Roman numerals of the categories correspond to the functional categories described in Fig 3. The percentage represents the proportion of proteins in each category.

According to their protein functions [26], the known proteins could be categorised into 5 classes. These functional classes included metabolism and energy production (33%; Class I), protein synthesis (7%; Class II), defence response (51%; Class III), cell division (2%; Class IV), and unclear classification (7%; Class V) (Figs 3 and 5). Among these classes, the most important category of defence response accounted for more than 50% of the total number of proteins identified.

### Differentially expressed proteins induced by the pathogen in susceptible (S) leaves

In total, 32 proteins were differentially expressed in susceptible leaves after inoculation with the *A*. *alternata* pathogen. According to their functional classifications, ten of these proteins were related to metabolism and energy production (Class I) (spots 8–9, 12, 14, 18, 20, 29 and 33–34), three were related to protein synthesis (Class II) (spot 30, 35 and 43), fifteen were related to defence response (Class III) (spots 1–2, 10–11, 13, 15, 19, 28, 31–32, 36–39 and 40), one was related to cell division (Class IV) (spot 6), and two were had unclear classifications (Class V) (spots 24–26) (Fig 3).

The largest group contained proteins related to defence response, including 2 up-regulated proteins (Fig 3, spots 11 and 13) and 13 down-regulated proteins (Fig 3, spots 1–2, 10, 15, 19, 28, 31–32, and 36–40). Interestingly, the two up-regulated proteins (spots 11 and 13) were annotated as the same protein, s-adenosylmethionine synthetase (SAMS), which serves as a precursor in polyamine biosynthesis [44]. In addition, three proteins were identified as the same heat-shock protein (spots 1–2 and 37), which is usually involved in the stress response.

Notably, a large portion of PR enzymes were critical enzymes involved in defence response, including beta-1,3-glucanase(spot 15), ascorbate peroxidase (APX) (spot 28), peroxiredoxin (spots 31–32), glutathione peroxidase (GPX) (spot 36), major allergen mal d 1(spots 38–39) and ribonuclease-like PR-10c (spot 40). Furthermore, ACC oxidase (spot 19), which catalyses the final reaction of the ethylene biosynthetic pathway [27], was down-regulated in susceptible leaves induced by the pathogen.

### Differentially expressed proteins induced by the pathogen in resistant (R) leaves

As shown in Fig 3, after inoculation with the *A*. *alternata* pathogen, 35 proteins were differentially expressed in resistant leaves. These proteins were classified into five groups based on their functions; eleven of them were related to metabolism and energy production (Class I) (spots 7, 9, 12, 14, 16, 18, 21–22, 33–34 and 42), two were related to protein synthesis (Class II) (spots 30 and 35), nineteen were related to defence response (Class III) (spots 1–5, 10–11, 13, 15, 17, 19, 23, 27 and 36–41), one was related to cell division (Class IV) (spot 6), and two had unclear classifications (Class V) (spots 24–25).

A total of 19 spots were identified as defence-related proteins, including 10 up-regulated proteins (spots 2–4, 23, 27, and 37–41) and 9 down-regulated proteins (spots 1, 5, 10–11, 13, 15, 17, 19, and 36). Notably, compared with susceptible leaves, SAMS (spots 11 and 13) exhibited opposite expression in resistant leaves and were all down-regulated after inoculation by the pathogen. In addition to these 2 proteins, heat-shock protein (Hsps; spots 2–5 and 37), major allergen mal d 1(spots 38–39 and 41), ribonuclease-like PR-10c (spot 40), peroxiredoxin (spot 27) and CXE carboxylesterase (spot 17) also exhibited differential expression patterns compared with those observed in susceptible leaves (Fig 3). Furthermore, several PR enzymes were also differentially expressed in resistant leaves, including beta-1,3-glucanase (spot 15), PR protein 8 (spot 23) and GPX (spot 36). These results indicate that during the course of inoculation, the host self-defence response mechanism is different in susceptible leaves and resistant leaves.

## Discussion

To our knowledge, there have been few comparative proteomic studies of the apple leaf response induced by *A*. *alternata*. In this study, we obtained an overview of the protein abundance changes that occur in apple leaves responding to an *A*. *alternata* infection. To elucidate differences between the response of resistant and susceptible seedlings to *A*. *alternata* infection, two different inoculation assays were conducted. Here, we discuss the possible response mechanisms that occur in apple leaves with pathogen infections.

### The responses of apple leaves toward *A*. *alternata* infection

A previous study revealed that the *A*. *alternata* apple pathotype causes Alternaria blotch in the apple host by producing a host-specific AM toxin [28]. Host plants usually use various defence mechanisms to protect themselves against infection by pathogens, including secretion of resistance-related proteins [29].

Chloroplasts are known to be important cellular organelles in green plants [30], and some studies have shown that chloroplasts can also serve as the primary site for the AM toxins [31]. In our study, few differences were identified in the chloroplasts of the control leaves (CK-S and CK-R) regardless of their number or shape. After infection with the *A*. *alternata* pathogen, the chloroplasts of susceptible leaves showed dramatic destruction evident from their shape and structure that was not observed in the resistant leaves. Based on the acting site of the AM toxins and known pathogenesis of the *A*. *alternata* pathogen [28], the results indicate that the pathogen may secrete AM toxins that act on the cells of the susceptible leaves, causing tissue damage. However, the resistant leaves may have certain antifungal activities that work against the *A*. *alternata* pathogen and are not present in the susceptible plants. Moreover, the following results from 2-DE analysis may provide critical biochemical evidence of the morphological changes in cell tissues that were observed under TEM.

### The metabolism and energy production related proteins

In our study, 33% of the protein spots identified were involved in metabolism and energy production, and more than half of these proteins were photosynthesis-related proteins. The differentially expressed photosynthetic proteins induced by the *A*. *alternata* pathogen in resistant and susceptible leaves may implicate light-sensing mechanisms in defence signalling. During the interaction between the pathogen and the plant host, a series of proteins related to photosynthesis were altered, suggesting a dynamic influence of the pathogen on the host photosynthetic machinery [32]. The up- and down-regulation of RuBisCO (Spot 33) in susceptible and resistant leaves may be related to the complicated defence response to the pathogen. The down-regulation of other photosynthesis proteins (Spot 18) during pathogen infection may indicate some feedback inhibition of photosynthetic genes [33].

### Defence-related proteins

In our study, 15 and 19 defence-related proteins were differentially expressed in susceptible and resistant leaves, respectively. In addition, 6 of 15 and 7 of 19 PR proteins were identified among these defence-related proteins, respectively.

Generally, host plants respond to pathogen attacks by producing a wide range of PR proteins [3]. PR proteins do not usually accumulate in healthy plants but are always induced by pathogen infection, improving the defensive capacity of host plants [34]. APX is the main enzyme of the ASC-GSH cycle and participates in the scavenging of potentially harmful H_2_O_2_ [35]. GPX is also an important enzyme involved in scavenging H_2_O_2_. In our study, these two proteins (Spots 28 and 36), belonging to the PR-9 family, were down-regulated in both susceptible and resistant leaves, indicating that antioxidant mechanisms may contribute to the antifungal mechanisms of the host plant against the *A*. *alternata* pathogen.

Beta-1,3-glucanase, a class-2 PR protein (PR-2), can protect host plants against pathogen infection by degrading the pathogen cell walls and contributing to pathogen death [36]. In our results, PR-2 (beta-1,3-glucanase) (Spot 15) decreased in abundance in both host plants after inoculation with the *A*. *alternata* pathogen. The expression pattern of beta-1,3-glucanase in apple leaves was similar to that in grapes infected with powdery mildew [37], although this phenomenon has not been observed in other host plants [33,38].

Hsps can play a crucial role in the response to pathogen infections to prevent host plant damage [39]. Of the Hsp family members, Hsp70 was confirmed to play a vital role in plant defence responses [40, 41]. In our results, six Hsps were differentially expressed in susceptible and resistant hosts, and 5 of them were high-molecular-weight Hsp70 (Spots 1–5 and 37). Based on previous observations [40–42] and our results, we speculate that Hsps may be involved in the initial stages of pathogen recognition and that different host cells may exhibit different Hsp expression profiles.

Major allergen mal d1 is an apple allergen and a member of the PR-10 class of proteins involved in the defensive capacity of host plants [43]. A previous study found that accumulated Mal d1 could induce apple fire blight [44]. In our research, three proteins were identified that were the same as mal d1 (Spots 38–39 and 41), and they exhibited differential expression patterns; all were down-regulated in susceptible leaves and up-regulated in resistant leaves, representing the differential anti-disease capacity of susceptible and resistant hosts.

SAMS is the key enzyme regulating polyamine synthesis and is also a key factor in the host plant response to pathogen challenge [45]. In our study, two proteins (Spots 11 and 13) exhibited differential expression in the 2 hosts; they were up-regulated in susceptible leaves and down-regulated in resistant ones, representing the differential anti-disease properties in diverse susceptible and resistant hosts.

### The other proteins

During the course of pathogen-host interaction, some basic physiologically regulated proteins were identified in the host plants, including those involved in protein synthesis and cell division and some of unclear classification [45]. In our study, proteins involved in these pathways were differentially regulated in response to *A*. *alternata* infection. Among them, the beta4 proteasome subunit (Spot 35) and an amino acid-binding protein (Spot 43) also exhibited differential expression in the two host plants, indicating that some common pathways are also involved in the antagonistic activity of susceptible and resistant hosts.

## Conclusion

To date, about *A*. *alternate* pathogen, several studies just focus on the identification, characterization, the disease cycle, and how to control the development of this pathogen [46–48]. A recent proteomic study also investigated the differential protein expression of transgenic mint infected by *A*. *alternate* pathogen [49].

In our present study, the high percentage of pathogen-responsive proteins signifies the necessity for studying the pathogen-responsive proteomes of apple plants during *A*. *alternata* pathogen infection, which are thought to be related to resistance. Thus, we compared the disease symptoms of susceptible and resistant host leaves inoculated with the *A*. *alternata* pathogen and investigated the molecular mechanisms of the host-pathogen interactions using a comparative proteomic approach. Our results suggest that beta-1,3-glucanase, APX, GPX and Hsps identified in the proteomes of susceptible and resistant hosts may play important roles in the anti-disease function of apple leaves in response to pathogen attacks. In addition, the PR protein mal d1 was down-regulated in the susceptible host but up-regulated in the resistant host, indicating that different levels of this factor may contribute to the noticeable antagonistic activity of resistant leaves against the *A*. *alternata* pathogen.

Overall, our study identified proteomic profiles in susceptible and resistant apple leaves that were induced by the *A*. *alternata* pathogen. The antifungal mechanisms of the apple host against the *A*. *alternata* pathogen may involve PR proteins and other defence response-related proteins. However, due to the complexity of the interaction between apple leaves and the *A*. *alternata* pathogen, it will also be of interest to define key factors contributing to the changes in the identified proteins. Therefore, further investigation will lead to more valuable insights into the molecular mechanisms of *A*. *alternate* suppression.

## Supporting Information

S1 Dataset The detailed information of Mascot searches for each protein. (XLSX)

S1 Table Differentially expressed proteins identified from resistant (R) and susceptible (S) leaves. (Machine readable version of Fig 3)
(DOCX)
