# Supplementary material for: Proteome Analysis of Pathogen-Responsive Proteins from Apple Leaves Induced by the Alternaria Blotch Alternaria alternata
Source: PLoS One. 2015 Jun 18;10(6):e0122233. doi: 10.1371/journal.pone.0122233 (PMC4472855; doi:10.1371/journal.pone.0122233)
Supplement: S1 Table — (Machine readable version of Fig 3) (DOCX) [file pone.0122233.s002.docx]

**S1_Table. Differentially expressed proteins identified from resistant (R) and susceptible (S) leaves**

| **Spot No.** | **Spot % volume variation** | **Mr (kDa) /pI** | **Accession**  **No.** | | **Description** | **Functional**  **categories** | | **Protein Score** | **Protein Score C.I.%** | **Pep Count.** | **Species** |
| --- | --- | --- | --- | --- | --- | --- | --- | --- | --- | --- | --- |
| **Metabolism and energy production** | | | | | | | | | | | |
| **7** |  | 55.44/5.09 | gi\|346683279 | | ATP synthase CF1  alpha subunit | | Ι | 192 | 100 | 6 | *Pyrus pyrifolia* |
| **8** |  | 52.10/5.02 | gi\|7708676 | | ATP synthase beta subunit | | Ι | 262 | 100 | 2 | *Thunbergia coccinea* |
| **9** |  | 56.89/7.1 | gi\|295684201 | | ADP glucose pyrophosphorylase small subunit 1-likeprotein | | Ι | 349 | 100 | 8 | *Malus x domestica* |
| **12** |  | 43.84/6.11 | gi\|211906520 | | UDP-D-apiose/UPD-D-xylose synthetase | | Ι | 140 | 100 | 7 | *Gossypium hirsutum* |
| **14** |  | 50.75/6.36 | gi\|297816078 | | glutamate-1-semialdehyde 2,1-aminomutase 2 | | Ι | 121 | 100 | 5 | *Arabidopsis lyrata subsp. lyrata* |
| **16** |  | 42.83/6.06 | gi\|118175929 | | chloroplast sedoheptulose-1,7-bisphosphatase | | Ι | 306 | 100 | 8 | *Morus alba var. multicaulis* |
| **18** |  | 97.45/6.4 | gi\|55585585 | | phosphoribulokinase-like protein 2 | | Ι | 171 | 100 | 1 | *Elaeis guineensis* |
| **20** |  | 30.86/6.2 | gi\|60101357 | | glutamine synthetase | | Ι | 167 | 100 | 3 | *Vigna radiata* |
| **21** |  | 37.04/7.06 | gi\|211906470 | | glyceraldehyde-3-phosphate dehydrogenase | | Ι | 344 | 100 | 9 | *Gossypium hirsutum* |
| **22** |  | 41.09/7.98 | gi\|41052915 | | putative ferredoxin-NADP(H) oxidoreductase | | Ι | 321 | 100 | 11 | *Oryza sativa Japonica Group* |
| **29** |  | 27.37/5.24 | gi\|414550 | | cytosolic triose phosphate isomerase | | Ι | 150 | 100 | 4 | *Arabidopsis thaliana* |
| **33** |  | 26.79/7.01 | gi\|9909636 | | ribulose 1,5-bisphosphate carboxylase | | Ι | 137 | 100 | 5 | *Elatine hydropiper* |
| **34** |  | 24.60/8.45 | gi\|315364830 | | chloroplast Rieske-type iron-sulfur protein | | Ι | 155 | 100 | 6 | *Citrullus lanatus* |
| **42** |  | 51.99/5.91 | gi\|75758075 | | carboxylase/oxygenase large subunit | | Ι | 406 | 100 | 20 | *Eryngium giganteum* |
| **Protein synthesis** | | | | | | | | | | | |
| **30** |  | 33.97/6.69 | gi\|170131 | | ribosomal protein 30S subunit | ΙΙ | | 114 | 100 | 6 | *Spinacia oleracea* |
| **35** |  | 14.59/6.4 | gi\|14594929 | | putative beta4 proteasome subunit | ΙΙ | | 182 | 100 | 4 | *Nicotiana tabacum* |
| **43** |  | 30.92/5.48 | gi\|255564051 | | amino acid-binding protein, putative | ΙΙ | | 180 | 100 | 3 | *Ricinus communis* |
| **Defense response** | | | | | | | | | | | |
| **1** |  | 71.52/5.17 | gi\|359486799 | | heat-shock cognate 70 kDa proteinisoform 2 | ΙΙΙ | | 921 | 100 | 19 | *Vitis vinifera* |
| **2** |  | 71.57/5.17 | gi\|6969976 | | high-molecular-weight heat-shock protein | ΙΙΙ | | 763 | 100 | 18 | *Malus xdomestica* |
| **3** |  | 72.04/5.27 | gi\|186898205 | | heat-shock protein 70 | ΙΙΙ | | 436 | 100 | 13 | *Hevea brasiliensis* |
| **4** |  | 72.04/5.27 | gi\|186898205 | | heat-shock protein 70 | ΙΙΙ | | 573 | 100 | 15 | *Hevea brasiliensis* |
| **5** |  | 72.04/5.27 | gi\|186898205 | | heat-shock protein 70 | ΙΙΙ | | 551 | 100 | 17 | *Hevea brasiliensis* |
| **10** |  | 43.68/5.83 | gi\|356539350 | | bifunctional polymyxin resistance protein ArnA-like | ΙΙΙ | | 204 | 100 | 5 | *Glycine max* |
| **11** |  | 43.61/5.5 | gi\|224108858 | | s-adenosylmethionine synthetase 3 | ΙΙΙ | | 411 | 100 | 12 | *Populus trichocarpa* |
| **13** |  | 43.61/5.5 | gi\|224101473 | | s-adenosylmethionine synthetase 1 | ΙΙΙ | | 487 | 100 | 13 | *Populus trichocarpa* |
| **15** |  | 21.76/9.21 | gi\|226789714 | | beta-1,3-glucanase | ΙΙΙ | | 153 | 100 | 3 | *Malus x domestica* |
| **17** |  | 41.02/5.24 | gi\|82697951 | | CXE carboxylesterase | ΙΙΙ | | 157 | 100 | 5 | *Malus pumila* |
| **19** |  | 36.53/5.28 | gi\|27372289 | | ACC oxidase | ΙΙΙ | | 130 | 100 | 5 | *Malus x domestica* |
| **23** |  | 18.42/4.53 | gi\|138753498 | | pathogenesis-related protein 8 | ΙΙΙ | | 124 | 100 | 2 | *Malus x domestica* |
| **27** |  | 22.21/4.92 | gi\|327422155 | | 2-cys-peroxiredoxin | ΙΙΙ | | 135 | 100 | 4 | *Vigna unguiculata* |
| **28** |  | 27.71/5.53 | gi\|145581388 | | ascorbate peroxidase | ΙΙΙ | | 155 | 100 | 5 | *Malus x domestica* |
| **31** |  | 25.05/8.37 | gi\|300078580 | | peroxiredoxin | ΙΙΙ | | 393 | 100 | 7 | *Jatropha curcas* |
| **32** |  | 21.96/4.93 | gi\|47027073 | | 2-cys peroxiredoxin-like protein | ΙΙΙ | | 139 | 100 | 4 | *Hyacinthus orientalis* |
| **36** |  | 18.69/6.14 | gi\|33308408 | | glutathione peroxidase | ΙΙΙ | | 507 | 100 | 12 | *Malus x domestica* |
| **37** |  | 18.11/5.54 | gi\|255558882 | | heat-shock protein, putative | ΙΙΙ | | 149 | 100 | 6 | *Ricinus communis* |
| **38** |  | 17.69/5.67 | gi\|4590376 | | major allergen mal d 1 | ΙΙΙ | | 163 | 100 | 6 | *Malus x domestica* |
| **39** |  | 17.69/5.67 | gi\|4590376 | | major allergen mal d 1 | ΙΙΙ | | 418 | 100 | 8 | *Malus x domestica* |
| **40** |  | 17.70/5.67 | gi\|15418742 | | ribonuclease-like PR-10c | ΙΙΙ | | 525 | 100 | 9 | *Malus x domestica* |
| **41** |  | 17.51/5.62 | gi\|2443824 | | major allergen Mal d 1 | ΙΙΙ | | 244 | 100 | 6 | *Malus x domestica* |
| **Cell division** | | | | | | | | | | | |
| **6** |  | 75.50/6.43 | gi\|255558698 | | cell division protein ftsH, putative | ΙV | | 880 | 100 | 13 | *Ricinus communis* |
| **Unclear classification** | | | | | | | | | | | |
| **24** |  | 38.07/6.51 | gi\|356545090 | uncharacterised protein | | V | | 107 | 100 | 1 | *Glycine max* |
| **25** |  | 26.34/5.29 | gi\|227204455 | AT2G37660 | | V | | 167 | 100 | 2 | *Arabidopsis thaliana* |
| **26** |  | 26.65/9.62 | gi\|226747930 | Mdfwg2100K20.g1 Apple_EST_ | | V | | 389 | 100 | 6 | *Malus x domestica* |

* Y axis: Relative expression of the spot (V%);

* X axis: column1: S-CK, 2: S-48h, 3: R-CK, 4: R-48h; CK: Control leaf; 48 h: Inoculation for 48 h

Data are representative of three independent biological replicates and given as intensity means ± S.D.
